# Assessing academic capacity and quality assurance in pharmacy education: a focus on the Eastern Mediterranean region

**DOI:** 10.1080/20523211.2025.2608552

**Published:** 2026-01-26

**Authors:** Zeinab Abedini, Ahmed Awaisu, Ian Bates, Banan Mukhalalati

**Affiliations:** a Clinical Pharmacy and Practice Department, College of Pharmacy, Qatar University, Doha, Qatar; b School of Pharmacy, University College London, London, UK

**Keywords:** Academic capacity, quality assurance, pharmaceutical workforce intelligence, pharmacy workforce, International-Pharmaceutical-Federation Development-Goals

## Abstract

**Background:**

Pharmacy workforce intelligence (PWI) involves the development, implementation, and evaluation of effective strategies and tools to ensure the availability and quality of pharmacy workforce (PW). Academic capacity (AC) is essential in producing graduates for PW, while quality assurance (QA) in education is crucial in developing competent PW. There is a lack of information about PWI in the Eastern Mediterranean Region (EMR). Based on the available data, there is a notable imbalance in PW distribution in EMR. This study aimed to evaluate the status of AC and QA of pharmacy education in the EMR using the International-Pharmaceutical-Federation (FIP)’s Development-Goals and their associated mechanisms as a framework.

**Methods:**

An explanatory sequential mixed-methods approach was used. The quantitative phase involved distributing a validated questionnaire among pharmacy leaders of all accessible pharmacy schools in the EMR. The qualitative phase involved the conduct of semi-structured interviews with pharmacy leaders, and the data were thematically analysed.

**Results:**

Of 112 identified pharmacy leaders, 61 participated in the survey (response rate, 55%) and 14 participated in the interviews. Most data were consistent among the quantitative and qualitative results. In both phases, most participants reported implementing a student – teacher ratio (70%), periodic accreditation (82%), adoption of global QA standards (67%), and involving key stakeholders in programme development. Enrolment planning based on workforce needs (51%) and capacity-building for teacher-practitioners (47%) were less common and less emphasised in interviews.

**Conclusion:**

Although some AC and QA mechanisms are achieved, many require further improvement. Policymakers could establish a national body representing PW, improve workforce data systems for evidence-based enrolment planning, invest in faculty development, and standardised QA frameworks. Standardising stakeholder engagement and enhancing graduate tracking would further ensure that programmes remain aligned with workforce and health system needs.

## Background

The pharmacy workforce (PW), which includes registered pharmacists, pharmacy technicians, pharmaceutical scientists, pharmacy support cadres, and pre-service students and trainees who operate in multiple settings with a diverse scope of practice, is an essential part of the healthcare team (Almarsdóttir & Traulsen, 2005; Anderson et al., 2010; Boniol et al., 2022; Hepler & Strand, 1990; Nkansah et al., 2010; Van den Bogert et al., 2012). The lack of adequately trained pharmacists hinders the provision of pharmaceutical services, contributing to disparities in access to medicines worldwide (Boniol et al., 2022; Campbell et al., 2013; Desselle, 2006; Diallo et al., 2003; El-Jardali et al., 2007; Fisher et al., 2022; Gu et al., 2021; Jenner et al., 2010; Liu et al., 2017; McIsaac et al., 2024; Narasimhan et al., 2004; *Quality Assurance of Pharmacy Education: The FIP Global Framework*, 2014; Rees, 2015). Recent international evidence suggests that the long-term development of the pharmacy workforce relies not only on the expansion of educational capacity but also on ensuring pharmacist satisfaction through effective leadership, equitable workloads, and opportunities for professional advancement (Anderson et al., 2008; Bader, Bates, Charman, et al., 2017; Barakat & Sallam, 2025; Behar-Horenstein et al., 2018; Mann et al., 2018; Rueben et al., 2020).

According to the International Pharmaceutical Federation (FIP) Goals 2021 Report, pharmacy workforce intelligence (PWI) is a national strategy that involves gathering and disseminating PW data, planning pharmacy skill mixes and advanced and specialised practices (*The FIP Development Goals Report, 2021: Setting Goals for the Decade Ahead*, 2022). The goal of PWI is ensuring the availability of competent PW, optimising health outcomes, and facilitating the achievement of global standards in pharmaceutical care, which are highly dependent on pharmacy education (Anderson et al., 2010; Bader, Bates, Schneider, et al., 2017; *Quality Assurance of Pharmacy Education: The FIP Global Framework*, 2014). According to the 2013 FIP Global Education Report, pharmacy education has differences in terms of academic scope, the infrastructure of institutions, and quality assurance (QA) mechanisms across different countries (*FIPEd Global Education Report*, 2013). Thus, FIP developed a QA framework for pharmacy education that can be applied across various educational contexts to ensure the delivery of high-quality education (*Global Vision for Education and Workforce*, 2016; *Pharmaceutical Workforce Development Goals*, 2016; *The FIP Development Goals Report*, 2021: *Setting Goals for the Decade Ahead*, 2022).

In 2016, the FIP developed a global strategy focusing on three primary pillars: ‘Global Vision for Education and Workforce’ (*Global Vision for Education and Workforce*, 2016), ‘Pharmaceutical Workforce Development Goals (PWDGs)’ (*Pharmaceutical Workforce Development Goals*, 2016), and ‘Nanjing Statements on Pharmacy and Pharmaceutical Sciences Education’ (*Nanjing Statements: Statements on Pharmacy and Pharmaceutical Sciences Education*, 2017). In 2021, FIP’s PWDGs were enhanced and expanded to include 21 goals, with the name; FIP Development Goals (FIP DGs) (*The FIP Development Goals Report*, 2021: *Setting Goals for the Decade Ahead*, 2022). The FIP DGs integrate ‘practice’, ‘science’, and ‘workforce and education’ component in each goal. Each development goal, along with its associated mechanisms, has been described with reference to these three components to ensure the comprehensiveness (*The FIP Development Goals Report*, 2021: *Setting Goals for the Decade Ahead*, 2022). The academic capacity development goal (AC) (DG1), which ensures accessibility and availability of the pharmaceutical workforce, and the quality assurance development goal (QA) (DG3), which ensures the production of competent pharmacists, are high priority development goal because they relate to pharmacy education (Enemark & Williamson, 2004; *Quality Assurance of Pharmacy Education: The FIP Global Framework*, 2014; *The FIP Development Goals Report*, 2021: *Setting Goals for the Decade Ahead*, 2022). Although the pharmaceutical workforce development encompasses many components, the foundation of this development is pharmacy education (Anderson et al., 2008; Bader, Bates, Charman, et al., 2017; Bader, Bates, Schneider, et al., 2017; Behar-Horenstein et al., 2018; Mann et al., 2018).

There is a scarcity of evidence on the PWI in the Eastern Mediterranean Region (EMR) countries compared to other World Health Organization (WHO)’s regions (El-Jardali et al., 2007; Jenner et al., 2010; Rees, 2015; *WHO’s Strategy for the Eastern Mediterranean Region, 2020–2023: Turning Vision 2023 into Action*, 2019). This scarcity limits the ability to monitor healthcare systems and improve workforce quality, which potentially affect the region's capacity to deliver effective pharmaceutical services (El-Jardali et al., 2010). Moreover, the predicted risk of global shortage of pharmacists by 2030 hinder the delivery of essential pharmaceutical services and deepen global inequalities in access to medicines and qualified pharmacists in the region (Boniol et al., 2022; El-Jardali et al., 2007; *Quality Assurance of Pharmacy Education: The FIP Global Framework*, 2014). In addition, there is a lack reliable PW data in the EMR (Bates et al., 2016; Bates, John, Meilianti, et al., 2018; Bates, John, Seegobin, et al., 2018; El-Jardali et al., 2007;Mukhalalati, Ashour, et al., 2020) as well as disparities in the distribution of the PW. For instance, countries like Afghanistan, Pakistan, and Somalia have more pharmacies than pharmacists (Bates et al., 2016), while others, such as Egypt and Jordan, graduate the highest number of pharmacists globally (Bates, John, Meilianti, et al., 2018). These imbalances highlight the urgent need to explore the PW status in the region to facilitate future workforce planning and targeted interventions to ensure a fair and sustainable workforce distribution. Without accurate data, it is difficult to identify regional priorities, make informed decisions, and implement effective solutions to address workforce gaps in EMR.

The FIP DGs is an international framework to enhance pharmacy workforce development. One of the main aspects of developing pharmacy workforce is to focus on the development of pharmacy education. To the best of our knowledge, the status of AC and QA in pharmacy education by using FIP DGs (DG1 and DG3 and their respective mechanisms) remains unknown in the EMR. Besides, capacity building is a fundamental goal of FIP DGs; without a skilled and knowledgeable pharmacy workforce, there would be no competent pharmacy workforce to achieve FIP’s goals. Quality assurance within the academic setting is extremely important, as high-quality education is necessary for training competent pharmacists, which in turn positively influences pharmacy practice. Thus, focusing on the pharmacy education status in the EMR countries can enhance the pharmacy workforce development in the region, and it can contribute to the overall pharmacy workforce development. Although the quality of education and capacity building for pharmacy workforce among EMR countries is diverse, FIP DGs created overall development goals with flexibility that can be adapted in multiple settings. Given this pressing need, this study aims to evaluate the AC and QA of pharmacy education in the EMR using the FIP DGs as a framework.

## Methods

An explanatory sequential mixed-methods approach was conducted at Qatar University, which involved a quantitative phase followed by a qualitative one (Hadi & Closs, 2016; Robinson, 2007; Toyon, 2021). The study was approved by the Qatar University Institutional Review Board (QU-IRB 1781-E/22). Prior to participation, email invitations were sent by the principal investigator to all eligible pharmacy leaders. All participants were provided with adequate information about the study along with an informed consent form in the email invitation. The consent form included the study purpose, definition of main concepts, anticipated benefits and risks of participation in the study, anonymity, and confidentiality. For the quantitative phase, the email invitation contained a link to a self-administered online questionnaire hosted on SurveyMonkey®, along with the informed consent information. The introductory page of the survey required participants to click on the ‘Continue’ button if they agreed to participate and complete the survey. They were given the opportunity to contact the lead Principal Investigator and IRB committee in case of any concerns at any time during the project. Participation information was kept anonymous. The participant’s personal information was not asked throughout the survey; however, the pharmacy schools/colleges/programmes name was asked as an optional question. This anonymity and secure data handling were the strategies used to assure confidentiality.

For the qualitative phase, the email invitations were sent to all the eligible pharmacy leaders. The consent form and PIL were sent to participants before the interviews, and it was requested to sign the consent forms prior to conduction of the interviews. After the signed consent forms were returned electronically via emails, then the interviews were scheduled according to the availability of the participants. All information collected from the electronic questionnaire and interviews were stored on password-protected laptops with access to the research team only. All research materials would typically be stored for five years and then destroyed according to Qatar University’s standard operating procedures.

## Quantitative phase

### Population and sampling

A universal sampling approach was used which included pharmacy leaders in all pharmacy programmes in the EMR that offer at least BSc Pharmacy or PharmD degrees (N = 112). The email addresses were obtained from the pharmacy programmes official websites.

### Tool development and validation

A new questionnaire was developed using the FIP DGs as a framework. The questionnaire contained seven main sections with 75 items: demographic characteristics of the pharmacy programme (7 items); AC workforce (23 items); AC practice (14 items); AC science (9 items); QA workforce (8 items); QA practice (11 items); and QA science (3 items).

The content validity and face validity were conducted during the questionnaire development and validation. Furthermore, Cronbach’s Alpha test was conducted to determine the internal consistency reliability of the items after the tool was distributed among all the participants.

### Quantitative data collection

A cross-sectional study using a self-administered questionnaire was conducted through SurveyMonkey® (Survey Monkey Inc., San Mateo, California, USA). Invitation emails, which included a participant information leaflet (PIL), a consent form, and a link to the survey were sent to all eligible pharmacy programmes. The survey was open from (1 March to 31 August 2023) with reminders sent every 2–4 weeks. Due to long questionnaire (75 items) and the selection of study target population (accessible but busy pharmacy leaders), it was decided to send multiple reminders over 6 months period to reduce the non-response bias and enhance accuracy of the results.

### Quantitative data analysis

Data were analysed using IBM Statistical Package for the Social Sciences (SPSS for Windows version 28, IBM Corp, Armonk, NY, USA). Descriptive statistics such as frequencies (%) were used to describe pharmacy programme characteristics and other categorical variables. Moreover, inferential analysis was conducted to evaluate the association of governmental status, establishment year, and accreditation status of the pharmacy programme with all of the mechanisms of AC and QA goals. To assess associations, a chi-square test of independence was conducted for binary and categorical variables. When the assumptions were violated, the Fisher-Freeman-Halton exact test was conducted. Cramer’s V was used to interpret the strength of associations. For all statistical tests, an alpha level of 0.05 was used to determine statistical significance. For open-ended questions, a content analysis approach was used.

## Qualitative phase

### Population and sampling

A voluntary response sampling method was used to recruit participants in this phase from those who participated in the quantitative cross-sectional study. Of the 61 survey participants, 20 indicated interest to participate in the qualitative phase and provided their contact email addresses. Fourteen pharmacy education leaders were invited to participate in one-to-one semi-structured interviews. The consent form and PIL were sent to participants before the interviews.

### Qualitative data collection

A phenomenological approach (Lewis, 2015) was utilised to gain a deep understanding of the status of pharmacy education in the region, focusing on AC and QA goals. The social constructivist interpretative framework (Lewis, 2015; Winit-Watjana, 2016) was used to represent the knowledge and perceptions of the researcher. Semi-structured interviews allowed for flexibility while facilitating comparisons across respondents. The topic guide was developed using the FIP DGs as the framework and the results of the quantitative phase. Two researchers with expertise in pharmacy education reviewed and validated the guide to ensure its clarity, feasibility, and appropriateness for respondents.

The interviews were conducted in English between December 2023 and January 2024 via Microsoft Teams. Each interview session lasted 30–45 min and was recorded, transcribed verbatim, and reviewed to ensure accuracy and reliability. The interviews were conducted by ZA, who underwent training prior to the interviews. Participants were assigned alphanumerical codes for anonymity, and the data collection continued till data saturation was reached (Service, 2009).

### Qualitative data analysis

The transcripts were analysed thematically using the computer-assisted coding software, NVIVO®. The codes were created through a hybrid deductive/inductive coding approach. These codes were then compared and grouped into overarching themes and subthemes. Two researchers (ZA and BM) jointly conducted the analysis to ensure the validity and reliability of coding and themes generation, addressing any disparities through consensus. The Consolidated Criteria for Reporting Qualitative Research (COREQ) was followed (Tong et al., 2007).

### Data integration

Data were analysed separately based on the type (i.e. qualitative versus quantitative). However, the findings were combined in interpretation and presentation. As the study was a sequential mixed-methods design, the quantitative data were collected and analysed first. Then, they were used to create the topic guides for the qualitative phase. The AC and QA goals and mechanisms were organised and categorised based on the frequency and intensity of reporting, then they were discussed individually bringing evidence from both qualitative and quantitative data collected.

Since the study utilised a mixed-methods design, qualitative and quantitative data were collected to maximise the comprehensiveness of the results. The qualitative and quantitative data were collected separately and then they were merged in the interpretation and integration of the results. The perceived differences and similarities from both qualitative and quantitative data were complementary to each other and induced gaining a comprehensive understanding of AC and QA goals in the EMR countries.

## Results

### Quantitative phase

#### Demographic characteristics of the pharmacy programmes

Overall, 61 responses were collected out of 112 the eligible pharmacy programmes (response rate of 55%). The majority of the pharmacy programmes were from Jordan (15%), followed by Saudi Arabia (13.3%), Pakistan (13.3%), Iraq (11.7%), Egypt (10%), Lebanon (10%), United Arab Emirates (10%), Oman (6.7%), and Palestine (3.3%). Around 55% of the programmes were governmental, while 45% were private/semi-governmental. 53% of these programmes were established in 2000 or later, while the remainder were established between 1980 and 1999 (37%), and 1979 or earlier (10%). 71% of the pharmacy programmes offer BSc Pharmacy degree, 57% offer PharmD degree, 52% offer MSc in Pharmaceutical Sciences, and 38% offer PhD. degree. 82% of the programmes undergo periodic accreditation. Table 1 demonstrates the demographic characteristics of programmes.

**Table 1.** Demographic characteristics of the pharmacy programmes/schools/colleges (n = 61).

Characteristics	Frequency (%)
**Country of pharmacy programme/school/college**	
Jordan	9 (14.8)
Pakistan	8 (13.1)
Saudi Arabia	8 (13.1)
Iraq	7 (11.5)
Egypt	6 (9.8)
United Arab Emirates	6 (9.8)
Lebanon	6 (9.8)
Oman	4 (6.6)
Palestine/ Occupied Palestinian territory	2 (3.3)
Kuwait	2 (3.3)
Bahrain	1 (1.6)
Qatar	1 (1.6)
Libya	1 (1.6)
**Governmental status of pharmacy programme/school/college**	
Public (i.e. governmental)	34 (55.7)
Private and/or semi-governmental	27 (44.3)
Year of establishment of pharmacy programme/school/college*	
≤ 1979	6 (10.0)
1980–1999	22 (36.7)
≥ 2000	32 (53.3)
Type of academic programme(s) offered^*†^	
BSc Pharmacy degree programme	44 (72.1)
PharmD degree programme	35 (57.4)
MSc in Pharmaceutical Sciences	32 (52.4)
Ph.D. degree programme	23 (37.7)
MSc in Clinical Pharmacy	22 (36.1)
Others	8 (13%)
**Does your pharmacy degree programme undergo periodic accreditation by an accreditation body/agency?**	
Yes	49 (80.3)
No	9 (14.8)
I am not sure	3 (4.9)
If yes to the previous question, what is the accrediting body/agency for your pharmacy degree programme(s)?^*†^	
Government body – Ministry of Education	31 (50.8)
Regulatory agency (e.g. Pharmacy Board, Pharmacists Council, etc.)	17 (27.9)
Government body – Ministry of Health	11 (18)
Private or non-governmental accrediting body	5 (8.2)
Others	18 (29.5)
How often does your programme go through an accreditation evaluation?*	
Every 1–3 years	17 (34.7)
Every 4–6 years	26 (53)
> 6 years	2 (4.1)
As needed	4 (8.2)
Frameworks used for QA of pharmacy education*	
FIP Global Framework	4 (33.6)
WHO standards	1 (8.1)
Others	7 (58.3)

Note: *Some missing data. ^†^This question was a ‘multiple options response’ type.

#### AC, the workforce, practice, and science aspects

Table 2 outlines the status of AC in relation to the workforce (23 items), the practice (14 items), and the science (8 items). The workforce aspect revealed that 31 pharmacy programmes (51%) reported planning students’ enrolment capacity based on local workforce needs. This information is typically obtained from the Ministry of Education, Ministry of Labour, and other relevant stakeholders. The undergraduate curriculum offers a range of potential career options, including community pharmacy (95%), hospital pharmacy (90%), industrial pharmacy (85%), clinical pharmacy (77%), research (72%), regulatory affairs pharmacy (68%), academic pharmacy (63%), administrative pharmacy (51%) (Table 2).

**Table 2.** Summary of the AC items related to W, *P*, S aspects (n = 61).

Aspect	Item	Frequency (%)
**Workforce**	Does your academic pharmacy programme/school/college decide about students’ enrolment capacity based on the workforce needs of your country?*	
	Yes	31 (51.7)
	No	23 (38.3)
	I am not sure	6 (10)
**Workforce**	Does your academic pharmacy programme/school/college have an experiential training programme that includes practicing pharmacists as preceptors or clinician educators?*	
	Yes	55 (93.2)
	No	3 (5.1)
	I am not sure	1 (1.7)
**Workforce**	**What are the potential career options that the undergraduate curriculum in your academic pharmacy programme/school/college prepares students for?^†^**	
	Community pharmacy	58 (95.1)
	Hospital pharmacy	55 (90.2)
	Industrial pharmacy	51 (83.6)
	Clinical Pharmacy	44 (77)
	Research	46 (75.4)
	Regulatory affairs pharmacy	41 (67.2)
	Academic pharmacy	38 (62.3)
	Administrative pharmacy	31 (50.8)
	Others	6 (9.8)
**Workforce**	**What are the potential career options that the postgraduate curriculum (Pharm D and/or MSc and/or PhD) in your academic pharmacy programme/school/college prepares students for?^†^**	
	Research	41 (67.2)
	Academic pharmacy	40 (65.6)
	Clinical Pharmacy	40 (65.6)
	Industrial pharmacy	39 (63.9)
	Hospital pharmacy	34 (55.7)
	Community pharmacy	33 (54.1)
	Regulatory affairs pharmacy	26 (42.6)
	Pharmacoeconomics	22 (36.1)
	Administrative pharmacy	22 (36.1)
	Pharmacogenomics	18 (29.5)
	Nuclear pharmacy	7 (11.5)
	Veterinary pharmacy	5 (8.2)
**Workforce**	**Which of the following disciplines/specialty areas represents the cohort of pharmacy faculty members in your institution?^†^**	
	Pharmaceutical/Medicinal Chemistry	58 (95.1)
	Clinical Pharmacy and Practice	57 (93.4)
	Pharmacology	55 (90.2)
	Pharmaceutics or Pharmaceutical Technology	53 (86.9)
	Pharmacoeconomics	29 (47.5)
	Pharmacogenomics	23 (37.7)
	Social and Administrative Pharmacy	23 (37.7)
	Others	12 (19.7)
**Workforce**	Does your academic pharmacy programme/school/college consider any national and/or international standards (e.g. FIP or UNESCO) in terms of student-teacher ratio?*	
	Yes	41 (69.5)
	No	7 (11.9)
	I am not sure	11 (18.6)
**Workforce**	Does your academic pharmacy programme/school/college consider any national or international standards in terms of resource needs (e.g. physical, human, and financial resources)?*	
	Yes	42 (72.4)
	No	11 (19)
	I am not sure	5 (8.6)
**Workforce**	Does your academic pharmacy programme/school/college have active partnership and cooperation with relevant stakeholders (e.g. healthcare institutions, professional pharmacy organisations, and patient advocacy groups) at national level?*	
	Yes	57 (98.4)
	No	0
	I am not sure	1 (1.6)
**Workforce**	Does your academic pharmacy programme/school/college have active partnership and cooperation with relevant stakeholders (e.g. healthcare institutions, professional pharmacy organisations, and patient advocacy groups) at international level?*	
	Yes	34 (58.6)
	No	18 (31.0)
	I am not sure	6 (10.4)
**Workforce**	Does your academic pharmacy programme/school/college have active interprofessional education with relevant stakeholders (e.g. colleges of medicine, nursing and other allied health) at national level?*	
	Yes	45 (77.6)
	No	10 (17.2)
	I am not sure	3 (5.2)
**Workforce**	Does your academic pharmacy programme/school/college have active interprofessional education with relevant stakeholders (e.g. colleges of medicine, nursing and other allied health) at international level?*	
	Yes	19 (32.8)
	No	35 (60.3)
	I am not sure	4 (6.9)
**Workforce**	Does your pharmacy programme/school/college have a well-established interprofessional education programme involving key stakeholders (e.g. colleges of medicine, nursing and other allied health) in your curriculum?*	
	Yes	38 (65.5)
	No	17 (29.3)
	I am not sure	3 (5.2)
**Workforce**	Does your academic pharmacy programme/school/college include formal mentorship programme for clinical academic educators (i.e. clinical faculty members)?*	
	Yes	34 (59.6)
	No	17 (29.8)
	I am not sure	6 (10.6)
**Workforce**	Does your academic pharmacy degree programme/school/college offer professional development activities in teaching and learning for clinical faculty members?*	
	Yes	47 (82.5)
	No	8 (14)
	I am not sure	2 (3.5)
**Workforce**	Does your academic pharmacy programme/school/college provide a clear career path guide to ensure career development for clinical faculty members?*	
	Yes	34 (58.6)
	No	14 (24.2)
	I am not sure	10 (17.2)
**Workforce**	Does your academic pharmacy degree programme/school/college offer professional development opportunities in research capacity enhancement for clinical faculty members?*	
	Yes	45 (77.6)
	No	9 (15.5)
	I am not sure	4 (6.9)
**Workforce**	Does your academic pharmacy degree programme/school/college collect data on current job placements of all its alumni/graduates?*	
	Yes	43 (74.1)
	No	7 (12.1)
	I am not sure	8 (13.8)
**Workforce**	Is there any existing database with relevant government sectors (e.g. Ministry of Health) or professional pharmacy bodies on number of graduates and different sectors that they work on the number of graduates and on the different sectors that they work at?*	
	Yes	29 (50)
	No	7 (12.1)
	I am not sure	22 (37.9)
**Workforce**	Does your academic pharmacy programme/school/college utilise relevant data and evidence to justify and support investment in pharmacy education?*	
	Yes	36 (64.3)
	No	9 (16.1)
	I am not sure	11 (19.6)
**Practice**	Does your pharmacy programme/school/college offer capacity building programmes for teacher-practitioners and in-practice education providers (e.g. preceptors for entry-to practice degree and advanced training) to enhance their experiential teaching and learning abilities?*	
	Yes	27 (48.2)
	No	19 (33.9)
	I am not sure	10 (17.9)
**Practice**	Does your pharmacy programme/school/college support or engage in developing in-service training such as residency or fellowship or specialisation for practicing pharmacists in the country?*	
	Yes	15 (26.8)
	No	30 (53.6)
	I am not sure	11 (19.6)
**Practice**	Do you have any educational frameworks or structures for practice-related postgraduate programmes (e.g. programmes with structured career pathway from internship to advanced practice or specialisation)?*	
	Yes	11 (20)
	No	30 (54.5)
	I am not sure	14 (25.5)
**Practice**	Does your pharmacy programme/school/college follow any accreditation standards and/or programme learning standards and/or competency standards for practice-related postgraduate programmes followed by the pharmacy programme/school/college?*	
	Yes	24 (42.9)
	No	24 (42.9)
	I am not sure	8 (14.3)
**Practice**	If yes to the previous question, please specify the accreditation standards and/or programme learning standards and/or competency standards for practice-related postgraduate programmes.*^†^	
	Accreditation Council for Pharmacy Education (ACPE)	11 (18)
	The American College of Clinical Pharmacy (ACCP)	8 (13.1)
	Canadian Council for Accreditation of Pharmacy Programmes (CCAPP)	5 (8.2)
	National Association of Pharmacy Regulatory Authorities (NAPRA)	4 (6.6)
	Model Standards of Practice for Canadian Pharmacists (MSOP)	0
	The European Association of Hospital Pharmacists (EAHP)	0
	Other (please specify)	8 (13)
**Practice**	Does your country have any structure/programme for in-practice interprofessional or interdisciplinary (multiple healthcare professionals) education and training?*	
	Yes	13 (23.2)
	No	23 (41.1)
	I am not sure	20 (35.7)
**Practice**	42. Do you have robust key performance indicators to evaluate the training performance/competencies within a variety of practice settings in your country?*	
	Yes	26 (46.4)
	No	8 (14.3)
	I am not sure	22 (39.3)
**Practice**	If your country has the performance indicators, who performs the training performance evaluation?*^†^	
	Clinical faculty members	17 (27.9)
	Multiple preceptors in a single training	15 (24.6)
	Main preceptor	13 (21.3)
	Student self-assessment	11 (18)
**Practice**	Do you have capacity building programmes for teacher practitioners and in-practice education providers (e.g. advanced practice preceptors) on performance evaluation and/or assessment for trainees in your country?*	
	Yes	15 (27.3)
	No	21 (38.2)
	I am not sure	19 (34.5)
**Science**	Are there national initiatives to communicate and collaborate between academic leaders and other key stakeholders (e.g. pharmaceutical industry and regulatory bodies) to determine the needs of pharmaceutical science education?*	
	Yes	31 (55.4)
	No	13 (23.2)
	I am not sure	12 (21.4)
**Science**	Are there any regional and/or international initiatives to communicate and collaborate between academic leaders and other key stakeholders (e.g. pharmaceutical industry and regulatory bodies) to determine the needs of pharmaceutical science education?*	
	Yes	15 (26.8)
	No	20 (35.7)
	I am not sure	21 (37.5)
**Science**	Does your academic pharmacy programme/school/college collaborate with relevant stakeholders and partners (e.g. major healthcare providers and industry) in designing and/or quality improvement of the curricular content in order to align with contemporary pharmaceutical sciences and practice?*	
	Yes	39 (69.6)
	No	8 (14.3)
	I am not sure	9 (16.1)
**Science**	If yes to the previous question, please specify the stakeholders and partners in designing and/or quality improvement of the curricular content in order to align with contemporary pharmaceutical sciences and practice.^*†^	
	The pharmaceutical industry	33 (54.1)
	Academic leaders	31 (50.8)
	Professional organisations	27 (44.3)
	Regulatory bodies	27 (44.3)
	Others (please specify)	6 (9.8)
**Science**	Does your pharmacy programme/school/college keep an inventory for training opportunities (national and international) in pharmaceutical sciences in order to broaden students’ access to curricular content?*	
	Yes	33 (58.9)
	No	13 (23.2)
	I am not sure	10 (17.9)
**Science**	If yes to the previous question, how do you keep the inventory for training opportunities (national and international) in pharmaceutical sciences accessible to students?^*†^	
	Shared networks/platforms	20 (32.8)
	Broadcasting email	15 (24.6)
	College webpage	15 (24.6)
	Others	6 (9.8)

Notes: *Some missing data. ^†^This question was a ‘multiple options response’ type.

Capacity-building programmes for teacher-practitioners and in-practice education providers were offered by 47% of the surveyed pharmacy programmes, while 35% reported not offering such programmes, and 18% were unsure of their situation (Table 2). The participating pharmacy programmes reported that 55% of them communicate/collaborate with stakeholders at the national level, while 26% do so at the regional or international level (Table 2). Furthermore, 71% of the pharmacy programmes collaborate with relevant stakeholders in designing and quality improvement of the curricular content.

#### QA, the workforce, practice, and science aspects

Table 3 presents the items assessing the status of QA in relation to the workforce (8 items), the practice (11 items), and the science (3 items). QA of education and training related to institutional infrastructure was achieved through various mechanisms such as internal academic programme review (70%), national accreditation process (68%), international accreditation process (53%), external academic programme review (47%), adaptation of professional association standards (22%), and none (2%). Stakeholders invited for input were identified as follows: faculty members (67%), students (58%), members of professional associations (53%), preceptors (43%), policymakers (37%), and others, such as college advisory committee (3%) and international consultants (2%), as presented in Table 3.

**Table 3.** Summary of the QA items related to W, *P*, S aspects (n = 61).

Aspect	Item	Frequency (%)
**Workforce**	How does your academic pharmacy programme/school/college ensure the quality of education and training in terms of institutional infrastructure?^*†^	
	Internal academic programme review	42 (68.9)
	National accreditation process	41 (67.2)
	International accreditation process	33 (54.1)
	External academic programme review	29 (47.5)
	Adaptation of professional association standards	13 (21.3)
	None	1 (1.6)
**Workforce**	How often does your programme perform the quality review?*	
	Every 1 year	21 (37.7)
	Every 2–3 years	10 (17.9)
	Every 4–5 years	21 (37.7)
	The programme does not perform quality review	2 (3.3)
**Workforce**	Does your academic pharmacy programme/school/college adopt/adapt global standards for QA of pharmacy education with consideration of local needs and practice?*	
	Yes	37 (67.3)
	No	7 (12.7)
	I am not sure	11 (20)
**Workforce**	Does your academic pharmacy programme/school/college implement transparent policies and procedures to ensure the quality of pharmacy education and training?*	
	Yes	51 (92.7)
	No	1 (1.8)
	I am not sure	3 (5.5)
**Workforce**	If yes to the previous question, are these policies and procedures of QA of pharmacy education accessible to key stakeholders?*	
	Yes	39 (78)
	No	4 (8)
	I am not sure	7 (14)
**Workforce**	Does your academic pharmacy programme/school/college invite input from critical stakeholder on the development of the educational and training components of the programme? (e.g. the curriculum or course syllabi, teaching, assessment, policies, etc.)*	
	Yes	43 (78.2)
	No	4 (7.3)
	I am not sure	8 (14.5)
**Workforce**	If yes to the previous question, who are the invited critical stakeholder to provide input on the development of the educational and training components of the programme (e.g. the curriculum or course syllabi, teaching, assessment, policies, etc.) educational and training components of the programme?^*†^	
	Faculty members	40 (65.6)
	Students	35 (57.4)
	Members of professional association	32 (52.5)
	Preceptors	26 (42.6)
	Policymakers	22 (36.1)
	Others	2 (3.8)
**Practice**	Does your country have clear standards of practice for pharmacists and/or pharmacy technicians/assistants practicing in hospital, community pharmacy, and/or other professional pharmacy sectors?*	
	Yes	38 (70.4)
	No	8 (14.8)
	I am not sure	8 (14.8)
**Practice**	Does your country ensure the delivery of pharmaceutical services in alignment with the healthcare system needs?*	
	Yes	35 (66)
	No	4 (7.6)
	I am not sure	14 (26.4)
**Practice**	Does your country have code of ethics/ethical standards for professional practice of pharmacy?*	
	Yes	43 (81.1)
	No	3 (5.7)
	I am not sure	7 (13.2)
**Practice**	If yes to the previous question, please select the responsible entity/ies for monitoring and ensuring the implementation of the code of ethics/ethical standards for professional practice of pharmacy.^*†^	
	Ministry of health	37 (60.7)
	Professional pharmacy body	23 (37.7)
	Higher administration of practice sector	7 (11.5)
	National health regulatory authority	1 (1.6)
**Practice**	Does your country have any mechanisms and/or indicators for quality improvement of collaborative practice in your pharmacy workforce setting?*	
	Yes	15 (30.6)
	No	6 (12.2)
	I am not sure	28 (57.1)
**Practice**	Does your country have any mechanisms and/or indicators for quality improvement of patient safety in your pharmacy workforce setting?*	
	Yes	27 (55.1)
	No	6 (12.2)
	I am not sure	16 (32.7)
**Practice**	Does your country have any mechanisms and/or indicators for quality improvement of professional standards in your pharmacy workforce setting?*	
	Yes	27 (54)
	No	5 (10)
	I am not sure	18 (36)
**Practice**	If yes to any of the previous questions (your country has mechanisms and/or indicators for quality improvement of collaborative practice/ patient safety/ professional standards in your pharmacy workforce setting), please specify the mechanisms and/or indicators for quality improvement.^*†^	
	Using pharmacovigilance systems	30 (49.2)
	Medication use reporting system	29 (47.5)
	Using technology	20 (32.8)
	Engaging in root-cause analysis	7 (11.5)
	None	5 (8.2)
	Old regulations	3 (4.9)
**Practice**	How does your country assure the education, training, performance, and professional development of the workforce, which results in ensuring the quality and effectiveness of pharmaceutical services?^*†^	
	Licencing exam	29 (47.5)
	Internship/apprenticeship programmes	22 (36.1)
	Mandatory continuing professional development (CPD) or continuing education (CE) for practicing pharmacists	20 (32.8)
	Proctored licensure exam	14 (23)
	Clinical attachment programmes	14 (23)
	Residency programmes	11 (18)
	None	5 (8.2)
**Practice**	What mechanisms does your country have for pharmaceutical service implementation and pharmaceutical service evaluation and monitoring?^*†^	
	Patient feedback	30 (49.2)
	Audit systems	22 (44.3)
	Health outcomes research	24 (39.3)
	Cost-effectiveness measures	19 (31.1)
	Health technology assessment	18 (29.5)
	None	9 (14.8)
**Science**	Are there any accessible guidance documents defining QA criteria for various pharmaceutical sciences focus areas (e.g. industry, bioequivalence, research and development for new therapeutic agents) in your country?*	
	Yes	23 (46)
	No	5 (10)
	I am not sure	22 (44)
**Science**	If yes to the previous question, can you please name the guidance documents used for the QA criteria for various pharmaceutical sciences focus areas in your country?^*†^	
	Standard Operating Procedures for QA (SOP)	18 (29.5)
	Good laboratory practice (GLP)	17 (27.9)
	Good manufacturing practice (GMP)	17 (27.9)
	Bioequivalence guidelines	17 (27.9)
	Policies and regulations of IRB	13 (21.3)
	Good clinical practice (GCP)	13 (21.3)
	The QA strategies from the Gulf Cooperation Councils (GCC) Pharmacy Congress	6 (9.8)
	FIP's QA framework	1 (1.6)
	Others	2 (3.3)
**Science**	Does your country collaborate with global and/or regional stakeholders to develop mechanisms aimed at reducing substandard and counterfeit medical and/or medicinal products?*	
	Yes	23 (46)
	No	2 (4)
	I am not sure	25 (50)

Notes: *Some missing data. ^†^This question was a ‘multiple options response’ type.

At the country level, 70% of the pharmacy programmes reported adherence to clear standards of practice for pharmacists and/or pharmacy technicians practicing in hospitals, community pharmacies, and other professional pharmacy sectors. Based on the perception of the participants, the alignment of pharmaceutical services with the healthcare system needs was reported by 66% of the programmes, 8% reported a lack of alignment, and 26% were unsure of their status (Table 3). The mechanisms and/or indicators for quality improvement in collaborative practice, patient safety, and professional standards were reported by 29%, 55%, and 54% of the pharmacy programmes, respectively (Table 3). The responses to the open-ended questions (13 items) related to AC and QA are summarised and presented in Appendix A.

#### Association assessments

Overall, the three aspects of governmental status, establishment year, and accreditation status of the pharmacy programme showed significant moderate associations with six items (Appendix B), four items (Appendix C), and four items (Appendix D), respectively. The governmental status was associated with the national or international standards for student-teacher ratio, active partnership and cooperation with relevant stakeholders at international level, data collection on current job placements of all its alumni/graduates, development of in-service training (e.g. residency/ fellowship/ specialisation) for practicing pharmacists in the country, and presence of robust key performance indicators to evaluate the training performance/competencies within a variety of practice settings. In addition, the establishment year of the pharmacy programme was associated with the presence of a well-established interprofessional education programme involving key stakeholders (e.g. colleges of medicine, nursing and other allied health) in the pharmacy programme curriculum, data collection on current job placements of all its alumni/graduates, implication of robust key performance indicators to evaluate the training performance/competencies within a variety of practice settings, and availability of any accessible guidance documents defining QA criteria for various pharmaceutical sciences focus areas. National or international standards for student-teacher ratio, national or international standards in terms of resource needs (e.g. physical, human, and financial resources), presence of active partnership and cooperation with relevant stakeholders (e.g. healthcare institutions, professional pharmacy organisations, and patient advocacy groups) at international level, and accessibility of the policies and procedures of QA of pharmacy education to key stakeholders were the 4 items moderately associated with the accreditation status of the pharmacy programme.

### Reliability

Cronbach’s alpha coefficient of the new scales is described in Appendix E. The overall internal consistency reliability was α = 0.85, which indicates good internal consistency of the items in the tool (*Cronbach’s Alpha in SPSS.*, n.d.; Taber, 2018; Tavakol & Dennick, 2011).

### Qualitative phase

Fourteen participants participated in the semi-structured interviews, representing a diverse range of countries, including Qatar, Palestine, Kuwait, Lebanon, Oman, UAE, Pakistan, KSA, Jordan, Egypt, and Iraq. Six main themes were identified through the semi-structured interviews, which are summarised in Table 4.

**Table 4.** Themes and subthemes that emerged from the semi-structured interviews.

Theme	Relevance to AC or QA	Subtheme(s)
T1. Factors affecting student enrolment experiences	AC	T1.1. Characteristics of the pharmacy programme T1.2. Resources influencing student enrolment in the pharmacy programme T1.3. Factors impacting students’ interest in programme enrolment
T2. The influence of the pharmaceutical workforce in shaping curricular planning	AC	T2.1. Means for assessing workforce local needs to plan the curriculum T2.2. Career opportunities for pharmacy graduates T2.3. Student-faculty ratio T2.4. Type of degree offered to graduates T2.5. Engagement of stakeholders in the curriculum development
T3. Teacher-practitioners learning experiences	AC	T3.1. Types of orientation provided to the preceptors T3.2. Mode of recruitment of preceptors T3.3. Facilitators aiding the improvement of experiential training
T4. Quality assurance strategies in pharmacy education	QA	T4.1. University-level strategies T4.2. National level strategies T4.3. International level strategies T4.4. QA of the practice settings
T5. Factors influencing the adoption of global standards	QA	T5.1. The attitude of authorities and faculty members regarding the adoption of global standards T5.2. Elements linked with the adoption of global standards T5.3. Availability of resources
T6. The significance of AC and QA in pharmaceutical workforce development	AC and QA	T6.1. The relation between AC and QA T6.2. The importance of AC T6.3. The importance of QA

#### T.1 factors affecting student enrolment

##### T1.1 Characteristics of the pharmacy programme

The key features of the pharmacy programmes included its governmental status and the composition of the students’ nationalities. For example, certain programmes prioritise admitting their nationals to empower their national workforce:

It is 100% governmental and mainly for Kuwaitis to empower the national workforce … P3 – Kuwait.

##### T1.2 Resources affecting student enrolment in the pharmacy programme

The participants identified several resources that influence student enrolment in their programmes. The budget constraint was mentioned as the main factor affecting students’ enrolment:

Although we are a public university, we did not receive any funding from the government in the last 10 years. So, we enroll as many students as we can to keep things running. P2 – Palestine.

##### T1.3 Factors impacting students’ interest in programme enrolment

Participants identified factors influencing students’ interest in enrolling in the programmes. These factors included gender, the political and economic stability of the country, the overall safety of residents, job opportunities, and programme promotion efforts. In many countries, there is a higher number of female students interested in pharmacy programmes compared to male students.

#### T.2 The influence of pharmaceutical workforce in shaping curricular planning

##### T2.1 Means for assessing workforce local needs to plan the curriculum

Local workforce needs are often not assessed before curriculum planning. As one participant noted:

We have not done a good homework in terms of looking at the supply and demand. P1 – Qatar.

##### T2.2 Career opportunities for pharmacy graduates

The pharmacy degree programmes in most EMR countries prepare students for a wide range of career opportunities. However, some participants acknowledged that recent graduates face limited career opportunities and expressed a desire to broaden their horizons.

The industrial pharmaceutical scope in Oman is not that big compared to neighboring countries and there is a plan for its expansion … P5 – Oman.

##### T2.3 Student–faculty ratio

Most programmes consider the student–faculty ratio as a main factor in curriculum planning and quality assessment. As one participant mentioned:

The ratio is one faculty member for every 25 students (1:25). P14 – Jordan.

##### T2.4 Type of degrees offered to graduates

Although most colleges offer a Bachelor of Pharmacy degree, some provide additional options such as a Diploma of Pharmacy Technology, PharmD, Master’s, and PhD degrees.

##### T2.5 Engagement of stakeholders in the curriculum development

Most programmes consider key stakeholders in the development of their curricular content, including the Ministry of Health, Ministry of Education, advisory boards, industrial CEOs or pharmaceutical industry representatives, hospital pharmacy representatives, community pharmacy owners, policymakers.

We involve employers like owners of community pharmacies, hospitals, industries, faculty members, Ministry of Health and Education, current students, and alumni. P6 – UAE.

#### T.3 teacher-practitioners learning experiences

##### T3.1 Types of orientation provided to the preceptors

Some participants highlighted the availability of structured orientation sessions or training to guide the teacher-practitioners during preceptorship. Others provide the preceptors with manuals or forms to support them throughout the training. However, some programmes do not offer any guidance materials.

##### T3.2 Mode of recruitment of preceptors

This subtheme explores whether preceptors are formally recruited by the university or voluntary providing the preceptorship service. As one participant indicated:

A good portion of my preceptors are hired by us; so, they are precepting 80% of their time. P6 – UAE

##### T3.3 Facilitators of experiential training

Several facilitators were identified to improve experiential training; those include student feedback for preceptors, student assessment, and continuous professional development (CPD) for preceptors.

#### T.4 QA strategies in pharmacy education

##### T4.1 University-level strategies

The majority of programmes incorporate faculty evaluation, quality assurance units, and student feedback as key sources of QA. Other pharmacy programmes adhere to key performance indicators and competency frameworks. One participant explained:

Firstly, students are allowed to assess the content and the assessments. Secondly, we have peer teaching evaluations. The third is a continuous evaluation from either the Head of the Department or the Associate Dean of Academics. P1 – Qatar

##### T4.2 National-level strategies

National accreditation standards, ministries regulations, and national licencing exams are the strategies used to evaluate the quality of pharmacy education at the national level. The national licencing exam was reported as a compulsory requirement for practice in some countries. However, other countries have no licencing exams.

We do not have a national exam; graduates join the workforce directly. P3 – Kuwait

##### T4.3 International-level strategies

Most programmes have obtained international accreditation or certification, while others have undergone external academic programme reviews. One participant explained:

The program is in affiliation with West Virginia University in the United States of America, and they do our major review with the national stakeholders. P5 – Oman

##### T4.4 QA of the practice settings

Most participants were not aware of the QA measures in the practice settings. Participants noted that high-quality education should ideally translate into high-quality pharmaceutical services. In that regard, a participant said:

I do not believe that there is a link. There is a CPD program for pharmacists, but there are no standards for the practice settings. P11 – Jordan

#### T.5 Factors influencing the adoption of global standards

##### T5.1 Attitude towards the adoption of global standards

In general, most participants reported no resistance and expressed a positive attitude towards adopting global standards (e.g. WHO, FIP, American Pharmacy Association) among national authorities and faculty members.

##### T5.2 The adoption of global standards

Some participants cited a lack of awareness of the existing global standards. However, most participants were well-informed and had plans for conducting needs assessments for adopting global standards. The participants expressed opposing perspectives regarding the adoption of global standards. An interviewee highlighted:

The pharmacy education is ahead of the pharmacy practice, so we need to improve the rules to be able to perform global standards. P7 – Oman

There are difficulties in understanding and accepting the role of clinical pharmacists, and physicians feel threatened. P8 – Pakistan

##### T5.3 Availability of resources

The pharmacy education leaders believed that the required resources for adopting global standards include space, budget, faculty members, practice sites for training, and policies related to the roles of the PW.

#### T.6 The significance of AC and QA in pharmaceutical workforce development

##### T6.1 The relation between AC and QA

Participants highlighted that AC and QA of pharmacy education are interconnected, emphasising that both are essential for developing an effective and high-quality PW. A participant noted:

Education provision cannot be disconnected from practice. We will always need them, and vice versa … P5 – Oman

##### T6.2 The importance of AC

This subtheme emphasises the importance of planning for AC, including faculty members, education, and curricular content. A participant explained that:

We need to provide the right resources from the infrastructure, classrooms, technology, and library, and the student affairs office. All resources need to be finetuned to ensure the preparing graduates for the practice. P10 – Lebanon

##### T6.3 The importance of QA

QA in pharmacy education plays an important role in producing a high-quality and competent pharmacy workforce. Participants highlighted the importance of maintaining strict QA measures to ensure programme effectiveness.

It is very important to have strict quality control criteria to maintain and enhance the program; especially for healthcare providers. P11 – Jordan

## Discussion

### Data integration

The integration of quantitative and qualitative data is detailed in Table 5. This study identified key factors influencing pharmacy school enrolment, including the school’s geographical location, programme fees, potential job opportunities, resource availability, political/economic stability, and applicant characteristics such as nationality, and gender. The data integration illustrated that some of the governmental schools that lacked governmental support and private/semi-governmental schools had to increase student enrolment to address financial constraints. Similarly, a study conducted in Malaysia suggested that better facilities, such as libraries, laboratories, and classrooms can facilitate students’ academic achievements and can increase a school's appeal to prospective students (Ramli & Mohd Zain, 2019).

**Table 5.** Integration of quantitative and qualitative findings.

Related to academic capacity or quality assurance	Quantitative		Qualitative		
	item	Percentage	theme	Subtheme(s)	Quote
Academic Capacity	Governmental status of the programme	Public(i.e. governmental): 55% Private and/or semi-governmental: 45%	T1. Factors affecting student enrolment experiences	T1.1. Characteristics of the pharmacy programme	‘It is 100% governmental and mainly for Kuwaitis, with very few expats as students’ P3 (Kuwait).
Academic Capacity	Does your academic pharmacy programme/school/college consider any national or international standards in terms of resource needs (e.g. physical, human, and financial resources)?*	Yes: 72% No: 19% I am not sure: 9%	T1. Factors affecting student enrolment experiences	T1.2. Resources influencing student enrolment in the pharmacy programme	‘We rely on the funding from the government, even being private universities. We rely a lot on scholarships given to us by the ministries. Imagine that a point in time will come if such funding is stopped, then that will affect the functioning of some of the colleges, especially for those who are not able to get students and do not think about sustainability. So I would say sustainability is always a challenge’ P5 (Oman). ‘Unfortunately, we do not have a limit for accepting students due to political and financial challenges. We are a government school but lack governmental support’ P2 (Palestine).
Academic Capacity	Does your academic pharmacy degree programme/school/college collect data on the current job placements of all its alumni/graduates?*	Yes: 74% No: 12% I am not sure: 14%	T1. Factors affecting student enrolment experiences	T1.3. Factors impacting students’ interest in the programme enrolment	‘The universities and schools must be responsible and accountable to not accept too many students. This will impact job opportunities and so on. Of course, another aspect of that is how we in academia make sure to create more job opportunities for our students with the knowledge, skills, [and] the right attitude so that they can compete in the job market. But at the same time, again, we should not flood the market with too many graduates’ P10 (Lebanon).
Academic Capacity	Does your academic pharmacy degree programme/school/college collect data on the current job placements of all its alumni/graduates?*	Yes: 74% No: 12% I am not sure: 14%	T2. Influence of the pharmaceutical workforce in shaping curricular planning	T2.1. Means for assessing workforce local needs to plan the curriculum	‘Unfortunately, we do not measure the need[s] of the market’ P14 (Jordan).
Academic Capacity	What are the potential career options that the undergraduate curriculum in your academic pharmacy programme/school/college prepares students for?^†^	Community pharmacy: 95% Hospital pharmacy: 90% Industrial pharmacy: 85% Clinical pharmacy: 77% Research: 72% Regulatory affairs pharmacy: 68% Academic pharmacy: 63% Administrative pharmacy: 51% Others: 10%	T2. Influence of the pharmaceutical workforce in shaping curricular planning	T2.2. Career opportunities for pharmacy graduates	‘The programme that is running in our college allows students to work in all the specialties in community pharmacies, in hospital pharmacy, in the clinical pharmacy department, the pharmaceutical industries as medical representatives, pharmacists in all of the departments, research and development, maybe the departments of dosage forms, quality control, and everything’ P12 (Egypt).
Academic Capacity	Does your academic pharmacy programme/school/college consider any national and/or international standards (e.g. FIP or UNESCO) in terms of student-teacher ratio?*	Yes: 69% No: 12% I am not sure: 19%	T2. Influence of the pharmaceutical workforce in shaping curricular planning	T2.3. Student-faculty ratio	‘Yes, it is based on higher education. I think we should have one faculty member for every 25 students. It is 1:25’ P14 (Jordan).
Academic Capacity	Type of academic programme(s)^*†^	BSc Pharmacy programme: 72% PharmD programme: 57% MSc in Pharmaceutical Sciences: 52% PhD programme: 38% MSc in Clinical Pharmacy: 36% Others: 13%	T2. Influence of the pharmaceutical workforce in shaping curricular planning	T2.4. Type of degree offered to graduates	‘[We have] Bachelor of Science in Pharmacy, Pharm D, Masters in Pharmaceutical Sciences, Masters in Clinical Pharmacy and Practice, and PhD programmes’ P1 (Qatar).
Academic Capacity	A. Please specify the stakeholders and partners in designing and/or improving the quality of the curricular content in order to align with contemporary pharmaceutical sciences and practice.^*†^	The pharmaceutical industry: 55% Academic leaders: 52% Professional organisations: 45% Regulatory bodies: 45% Others (please specify): 10%	T2. Influence of the pharmaceutical workforce in shaping curricular planning	T2.5. Engagement of stakeholders in the curriculum development	‘We focus on students and also employers, like owners of community [pharmacies and] hospitals. Industry [leaders] and faculty members are involved. Ministry of Health and Education, current students, and alumni are involved’ P6 (UAE). ‘We have an Advisory Council, and … the members of the Advisory Council are members of the faculty, like the dean, the assistant deans, the head of the department, as well as representatives from different sectors of the work feed or the stakeholders. So yes, this is how we communicate and how we have these regular discussions at least once each semester with the Advisory Council’ P11 (Jordan).
	B. Who are the invited critical stakeholder to provide input on the development of the educational and training components of the programme (e.g. the curriculum or course syllabi, teaching, assessment, policies, etc.) educational and training components of the programme?^*†^	Faculty members: 67% Students: 58% Members of professional associations: 53% Preceptors: 43% Policy makers: 37% Other: 3%			
Academic Capacity	Does your pharmacy programme/school/college offer capacity-building programmes for teacher practitioners and in-practice education providers (e.g. preceptors for entry-to-practice degrees and advanced training) to enhance their experiential teaching and learning abilities?*	Yes: 48% No: 35% I am not sure: 18%	T3. Teacher practitioners’ learning experiences	T3.1. Types of orientation provided to the preceptors	‘There are no orientation sessions or workshops for the preceptors’ P8 (Pakistan).
Academic Capacity	Do you have capacity-building programmes for teacher practitioners and in-practice education providers (e.g. advanced practice preceptors) on performance evaluation and/or assessment for trainees in your country?*	Yes: 27% No: 38% I am not sure: 35%	T3. Teacher practitioners’ learning experiences	T3.3. Facilitators aiding the improvement of experiential training	‘We also have professional development with the different activities related to teaching. Throughout the year for preceptors, we have four modules that the preceptors can take and help them to become better preceptors for the students’ P10 (Lebanon).
Quality Assurance	How does your academic pharmacy programme/school/college ensure the quality of education and training in terms of institutional infrastructure?^*†^	Internal academic programme review: 70% National accreditation process: 68% International accreditation process: 53% External academic programme review: 47% Adaptation of professional association standards: 22% None: 2%	T4. Quality assurance strategies in pharmacy education	T4.1. University-level strategies T4.2. National-level strategies T4.3. International-level strategies	‘They consider the ACPE [Accreditation Council for Pharmacy Education] and the national standards as the required evaluation’ P11 (Jordan). ‘Our BPharm and PharmD programmes have CCAPP [accreditation]. For graduate studies and research, we had the academic programme review for our master’s programme in 2016. So we invited two professors, one from the US and one from Canada, to evaluate our Master’s programme. Now, it is due, and we will invite two experts again, as there is no accreditation or certification for graduate programmes. They will be coming in 2024 to evaluate our master’s programme. For the PharmD and the bachelor degree, we obtained accreditation from [the] CCAPP this year up to 2028’ P1 (Qatar).
Quality Assurance	Does your country have clear standards of practice for pharmacists and/or pharmacy technicians/assistants practicing in hospitals, community pharmacies, and/or other professional pharmacy sectors?*	Yes: 70% No: 15% I am not sure: 15%	T4. Quality assurance strategies in pharmacy education	T4.4. Quality assurance of the practice settings	‘About the industry, they are following lab standards. But I am not sure about the hospitals, and I do not know actually what they follow. Because we have different quality of hospitals, and there are private hospitals and public hospitals, I cannot exactly answer this question’ P13 (Iraq).
Quality Assurance	Does your academic pharmacy programme/school/college adopt/adapt global standards for the quality assurance of pharmacy education with consideration of local needs and practice?*	Yes: 67% No: 13% I am not sure: 20%	T5. Factors influencing the adoption of global standards	T5.2. Elements linked with the adaptation of global standards	‘The issue is if it is something that does not make sense for Kuwait, then that would be the main barrier to not implement it’ P3 (Kuwait). ‘There are difficulties in understanding and accepting the role of clinical pharmacists, and physicians feel threatened. In hospitals, there is some sort of resistance to accepting new roles’ P8 (Pakistan). ‘Pharmacy education is ahead of the pharmacy practice, so we need to improve the rules to be able to perform by global standards’ P7 (Oman).

Notes: *Some missing data. ^†^This question was a ‘multiple option response’ type.

Many participants reported that most pharmacy graduates preferred clinical career opportunities over jobs in the pharmaceutical industries which can create imbalance in the pharmacy workforce. According to a study conducted in Saudi Arabia, 83.2% of respondents identified working as a hospital clinical pharmacist as their most desired career path (Alhaddad, 2018). Similarly, a qualitative case study conducted in Qatar indicated that working in hospitals and academia were the primary career aspirations of pharmacy students (Mukhalalati, Ashour, et al., 2020). Another study indicated a greater proportion of females compared to males in the PW globally, with some WHO regions, including the Americas, Europe, and the Western Pacific, displaying a female representation of more than 65% (Bates, John, Seegobin, et al., 2018).

Although 74% of pharmacy schools reported collecting data on current job placements for their alumni, most universities admitted lacking accurate data on the national demand for pharmacists in the semi-structured interviews. A study on the PW in the EMR highlighted an increasing number of pharmacy graduates in the region, but noted a lack of adequate workforce planning (Mukhalalati, Ashour, et al., 2020). Strengthening PWI through effective professional pharmacy bodies and policymakers could address this issue (Mukhalalati, Bader, et al., 2020). Thus, local workforce needs should be systematically collected and considered in curricular development to prevent potential career crises in the PW (Anderson et al., 2010; Arakawa et al., 2020; *FIPEd Global Education Report*, 2013; Lebovitz & Eddington, 2019).

The study suggested that most countries need to develop and improve structured orientation sessions for preceptors. A scoping review in 2020 highlighted the need for adopting a structured and evidence-based approach to preceptor training (Knott et al., 2020). Programmes should be educationally robust, practically oriented, and flexible to accommodate the varying needs of preceptors across diverse practice settings. Studies conducted in Lebanon (Zeitoun et al., 2020) and Qatar (Mukhalalati et al., 2022) recognised the crucial roles of preceptors and created a five-module interactive workshop to support preceptor development.

Overall, 82% of the pharmacy programmes undergo periodic accreditation, which is consistent with the data available regionally (Alkhateeb et al., 2018) and globally (Engle et al., 2023). A study conducted in the GCC countries found that international pharmacy accreditation/certification is more prevalent than national accreditation (Alkhateeb et al., 2018). While 70% of the participants reported adhering to clear pharmacy practice standards, many interviewees reported a lack of national practice standards in their settings. Aligning pharmacy practice standards with pharmacy education can have several positive outcomes. Therefore, pharmacy practice standards should be developed, tailored to national needs, and integrated into pharmacy education (Bader, Bates, Schneider, et al., 2017; *Quality Assurance of Pharmacy Education: The FIP Global Framework*, 2014).

Outdated national policies were identified by some participants as the main gap between pharmacy practice and education. A case study conducted in Qatar highlighted the absence of a Qatar Pharmacy Association as a significant barrier to the development of pharmacy workforce-related policies (Mukhalalati et al., 2021). Similarly, the lack of professional pharmacy leaders and stakeholders’ involvement in addressing workforce challenges was illustrated in another EMR study (Mukhalalati, Bader, et al., 2020).

## Strengths and limitations

To the best of our knowledge, this is the first study to evaluate the status of AC and QA goals using the FIP DGs as a framework. The mixed-methods approach enhanced the objectivity and comprehensiveness of the data. Overall, the survey achieved a response rate of 55%, and the semi-structured interviews had 14 participants, which are relatively high compared to similar studies conducted in the region. Data from most EMR countries were captured in both phases of the study, where the semi-structured interviews provided the participants with adequate flexibility to express their views.

Despite its strengths, this study had some important limitations that need to be acknowledged. The study focused exclusively on AC and QA, excluding the other 19 FIP DGs. The academic goals (DG1 and DG3) are highly related to pharmacy education – as they connect the educational system to the workforce, which might limit the generalizability of the tool if the objective were to evaluate the status of pharmacy practice settings. Furthermore, the questionnaire included 75 items, which may be time-consuming for respondents, potentially affecting the response rate and causing non-response and recall biases. The interviews were conducted individually, which might have reduced the dynamic and flexibility of the discussions, as well as the richness of the data. However, due to the nature and sensitivity of the data, individual interviews were considered the most appropriate method for data collection which facilitated participants’ comfort and transparency. There is a risk of sampling bias as the target population was only pharmacy leaders, however, the most accurate data about the pharmacy education status could have been only obtained from this target population. Although the possibility of social desirability bias cannot be entirely excluded, measures such as ensuring respondent anonymity and using neutrally worded items were used to minimise its impact.

## Conclusion

In conclusion, it is evident that pharmacy programmes in the EMR vary regarding the implementation of AC (DG1) and QA (DG3) goals of the FIP DGs. This mixed-methods research is a benchmark study for the evaluation of pharmacy education status in EMR countries. Without the baseline data about the status of the FIP DGs, it is difficult to understand which goals have already been achieved or need improvement. Consequently, future research should be conducted to evaluate the status of all other FIP DGs in all WHO regions.

While certain AC and QA mechanisms are achieved and maintained adequately, many still need substantial enhancement. Policymakers could address these gaps by forming a national PW representative body, improving workforce data systems to guide enrolment decisions, and investing in faculty development and unified QA standards. Additionally, more consistent stakeholder engagement and strengthened graduate tracking systems would help ensure programmes remain aligned with labour-market and health system priorities (Appendices F and G).

Future studies should aim to design pharmacy practice standards that align with pharmacy education standards. National policies need to be updated to reflect the emerging roles of the pharmacy workforce. The formation of national professional bodies and national practice and educational standards should be addressed to ensure the cohesiveness between pharmacy education and practice at both national and international levels. A reassessment of the status of pharmacy education may be performed in the near future to observe the improvements and identify potential challenges.

## Appendices

**Appendix A.** Summary of open-ended questions related to academic capacity and quality assurance of FIP pharmaceutical development goals.

Summary of open-ended questions related to academic capacity and quality assurance of FIP pharmaceutical development goals (n = 61)	
Item	Open-ended responses
Specify the sectors from which your college/academic programme obtain the information about the workforce need to consider in students’ enrolment capacity decision-making.*	Ministry of higher education Ministry of labour Stakeholders
Which national and/or international standards does your academic pharmacy programme/school/college consider in terms of student-teacher ratio?*	ACPE CCAPP Ministry of education National Authority for QA and Accreditation of Education (NAQAAE) UNESCO
Which national/international standards do you follow in terms of resources?*	ACPE CCAPP FIP Government resources Ministry of education
Which entity maintain the database about the number of graduates and different sectors that they work?*	College administration Graduate follow-up/Alumni committee Ministry of education Ministry of health Student affairs
Specify the capacity building programme of teacher-practitioners and in-practice education providers.*	CPD Structured orientation and training programmes
Specify the in-service training programme your pharmacy programme/school/college is supporting.*	PGY /Residency programmes Internship programmes
Name the kind of educational frameworks or structures for practice-related postgraduate programmes.*	National frameworks (e.g. Oman Qualification Framework, Saudi Commission for Health Specialties) National Institute of Health Specialties (NIHS) guided residency and fellowship programmes
Briefly describe the structure/programme and the key stakeholders/players for the in-practice interprofessional or interdisciplinary education and training.*	A training and education programmes for pharmacy organised by the Saudi Commission for Health Specialties Hospitals, industry, community pharmacy interdisciplinary education for drug research, translational research, residency for clinical pharmacy are offered in Shaukat Khanum Memorial Cancer Hospital and Research Centres and agha Khan hospital Interprofessional Education (IPE) Committee at Qatar University Health colleges level, engaging all health-related colleges in the country
Specify the name of the capacity building programme(s) for teacher-practitioners and in-practice education providers on performance evaluation, assessment, and feedback for trainees.*	Higher education commission announces such types of trainings time to time and asks nominations from public and private sectors both Preceptor training programme/ workshops
List the national initiatives or efforts to communicate and collaborate between academic leaders and other key stakeholders to determine the needs of pharmaceutical science education.*	Agreement between Ministry of Health and Ministry of Education Collaborations with the Jordanian Pharmacist Syndicate, JFDA, Ministry of Health (similar response for Kuwait, Lebanon, KSA, Oman) HEC Pakistan in collaboration with pharmacy council designed uniform curriculum system throughout the country Joint research projects with stakeholders Providing grants for research projects Pharmaceutical companies’ participation in developing curriculum (training, workshops)
List those regional and/or international initiatives to communicate and collaborate between academic leaders and other key stakeholders (e.g. pharmaceutical industry and regulatory bodies) to determine the needs of pharmaceutical science education in your country.*	Inviting national and international stakeholders to review programme Egyptian Drug Authority Collaboration of drug regulatory authorities with universities Collaborating with WHO, FIP, AACP, and colleges in US, UK, Australia, and New Zealand
Which global standards does your pharmacy programme/school/college adopt/adapt for QA of pharmacy education with consideration to local needs and practice? *	Accreditation Agency in Health and Social Sciences (AHPGS) Accreditation Council for Pharmacy Education standards FIP CCAPP WHO NAQAE
Name the standards of practice for pharmacists and/or pharmacy technicians/assistants practicing in hospital, community pharmacy, and/or other professional pharmacy sectors in your country.*	Standards of Saudi Central Board for Accreditation of Healthcare Institutes (CBAHI) Saudi Commission for Health Specialties Standards of Drug Regulatory Authority of Pakistan (DRAP) Standards of Egyptian Drug Authority (EDA) standards Standards of General Authority for Healthcare Accreditation and Regulation (GAHAR) in Egypt Standards of Jordan Pharmacists Association (JPA) Standards of Pharmacy Council of Pakistan (PCP), and Higher Education Commission (HEC) guidelines Standards of Lebanese Order of Pharmacists (OPL) Standards of Ministry of Public Health Standards of WHO

Note: *Some missing data.

**Appendix B.** Association between the governmental status of the pharmacy programme/school/college and the mechanisms of academic capacity and quality assurance goals.

Association between the governmental status of the pharmacy programme/school/college and the mechanisms of academic capacity and quality assurance goals			
Items	Frequency (%)		Association
Does your academic pharmacy programme/school/college consider any national and/or international standards (e.g. FIP or UNESCO) in terms of student-teacher ratio?	Governmental	Non-governmental	Freeman-Halton: 8.405 (*p*-value: 0.011) Cramer’s V: 0.375 (*p*-value: 0.012)
Yes	19 (55.9)	22 (88)	41 (69.5)
No	7 (20.6)	0	7 (12)
I am not sure	8 (23.5)	3 (12)	11 (18.5)
Does your academic pharmacy programme/school/college have active partnership and cooperation with relevant stakeholders (e.g. healthcare institutions, professional pharmacy organisations, and patient advocacy groups) at international level?	Governmental	Non-governmental	Freeman-Halton: 12.43 (*p*-value: 0.002) Cramer’s V: 0.466 (*p*-value: 0.002)
Yes	13 (39.4)	21 (84)	34 (58.6)
No	14 (42.4)	4 (16)	18 (31)
I am not sure	6 (18.2)	0	6 (10.3)
Does your academic pharmacy degree programme/school/college collect data on current job placements of all its alumni/graduates?	Governmental	Non-governmental	Freeman-Halton: 6.973 (*p*-value: 0.034) Cramer’s V: 0.355 (*p*-value: 0.026)
Yes	20 (60.6)	23 (92)	43 (74.1)
No	6 (18.2)	1 (4)	7 (12.1)
I am not sure	7 (21.2)	1 (4)	8 (13.8)
Does your pharmacy programme/school/college support or engage in developing in-service training such as residency or fellowship or specialisation for practicing pharmacists in the country?	Governmental	Non-governmental	Freeman-Halton: 10.892 (*p*-value: 0.005) Cramer’s V: 0.441 (*p*-value: 0.004)
Yes	4 (12.5)	11 (45.8)	15 (26.8)
No	18 (56.3)	12 (50)	30 (53.6)
I am not sure	10 (31.3)	1 (4.2)	11 (19.6)
Do you have robust key performance indicators to evaluate the training performance/competencies within a variety of practice settings in your country?	Governmental	Non-governmental	Freeman-Halton: 7.829 (*p*-value: 0.019) Cramer’s V: 0.362 (*p*-value: 0.031)
Yes	12 (37.5)	14 (58.3)	26 (46.4)
No	8 (25)	0	8 (14.3)
I am not sure	12 (37.5)	10 (41.7)	22 (39.3)
How often does your programme perform the quality review?	Governmental	Non-governmental	Freeman-Halton: 9.945 (*p*-value: 0.019) Cramer’s V: 0.437 (*p*-value: 0.018)
Others	2 (6.3)	0	2 (3.6)
Every 1 year	8 (25)	13 (54.2)	21 (37.5)
Every 2–3 years	9 (28.1)	1 (4.2)	10 (17.9)
Every 4–5 years	11 (34.4)	10 (41.7)	21 (37.5)
The programme does not perform quality review	2 (6.3)	0	2 (3.6)

**Appendix C.** Association between the establishment year of the pharmacy programme/school/college and the mechanisms of academic capacity and quality assurance goals.

Association between the establishment year of the pharmacy programme/school/college and the mechanisms of academic capacity and quality assurance goals				
Items	Frequency (%)			Association
Does your pharmacy programme/school/college have a well-established interprofessional education programme involving key stakeholders (e.g. colleges of medicine, nursing and other allied health) in your curriculum?	≤ 1979	1980–1999	≥ 2000	Freeman-Halton: 9.248 (*p*-value: 0.027) Cramer’s V: 0.309 (*p*-value: 0.04)
Yes	2 (33.3)	14 (70)	21 (67.7)	37 (64.9)
No	4 (66.7)	3 (15)	10 (32.3)	17 (29.8)
I am not sure	0	3 (15)	0	3 (5.3)
Does your academic pharmacy degree programme/school/college collect data on current job placements of all its alumni/graduates?	≤ 1979	1980–1999	≥ 2000	Freeman-Halton: 8.586 (*p*-value: 0.043) Cramer’s V: 0.306 (*p*-value: 0.029)
Yes	2 (33.3)	14 (70)	26 (83.9)	42 (73.7)
No	3 (50)	2 (10)	2 (6.5)	7 (12.3)
I am not sure	1 (16.7)	4 (20)	3 (9.7)	8 (14)
Do you have robust key performance indicators to evaluate the training performance/competencies within a variety of practice settings in your country?	≤ 1979	1980–1999	≥ 2000	Freeman-Halton: 9.833 (*p*-value: 0.026) Cramer’s V: 0.295 (*p*-value: 0.048)
Yes	3 (60)	4(20)	18 (60)	25 (45.5)
No	1 (20)	3(15)	4 (13.3)	8 (14.5)
I am not sure	1 (20)	13 (65)	8 (26.7)	22 (40)
Are there any accessible guidance documents defining QA criteria for various pharmaceutical sciences focus areas (e.g. industry, bioequivalence, research and development for new therapeutic agents) in your country?	≤ 1979	1980–1999	≥ 2000	Freeman-Halton: 9.584 (*p*-value: 0.025) Cramer’s V: 0.338 (*p*-value: 0.019)
Yes	4 (100)	7 (36.8)	12 (46.2)	23 (46.9)
No	0	0	5 (19.2)	5 (10.2)
I am not sure	0	12 (63.2)	9 (34.6)	21 (42.9)

**Appendix D.** Association between the accreditation status of the pharmacy programme/school/college and the mechanisms of academic capacity and quality assurance goals.

Association between the accreditation status of the pharmacy programme/school/college and the mechanisms of academic capacity and quality assurance goals				
Items	Frequency (%)			Association
Does your academic pharmacy programme/school/college consider any national and/or international standards (e.g. FIP or UNESCO) in terms of student-teacher ratio?	Have periodic accreditation	Do not have periodic accreditation	Not sure	Freeman-Halton: 13.734 (*p*-value: 0.002) Cramer’s V: 0.391 (*p*-value: 0.003)
Yes	37 (78.7)	4 (44.4)	0	41 (69.5)
No	5 (10.6)	2 (22.2)	0	7 (11.9)
I am not sure	5 (10.6)	3 (33.3)	3 (100)	11 (18.6)
Does your academic pharmacy programme/school/college consider any national or international standards in terms of resource needs (e.g. physical, human, and financial resources)?	Have periodic accreditation	Do not have periodic accreditation	Not sure	Freeman-Halton: 8.283 (*p*-value: 0.48) Cramer’s V: 0.349 (*p*-value: 0.022)
Yes	35 (76.1)	6 (66.7)	1 (33.3)	42 (72.4)
No	9 (19.6)	2 (22.2)	0	11 (19)
I am not sure	2 (4.3)	1 (11.1)	2 (66.7)	5 (8.6)
Does your academic pharmacy programme/school/college have active partnership and cooperation with relevant stakeholders (e.g. healthcare institutions, professional pharmacy organisations, and patient advocacy groups) at international level?	Have periodic accreditation	Do not have periodic accreditation	Not sure	Freeman-Halton: 13.906 (*p*-value: 0.002) Cramer’s V: 0.348 (*p*-value: 0.010)
Yes	32 (69.6)	1 (11.1)	1 (33.3)	34 (58.6)
No	10 (21.7)	7 (77.8)	1 (33.3)	18 (31)
I am not sure	4 (8.7)	1 (11.1)	1 (33.3)	6 (10.3)
If yes to the previous question, are these policies and procedures of QA of pharmacy education accessible to key stakeholders?	Have periodic accreditation	Do not have periodic accreditation	Not sure	Freeman-Halton: 11.079 (*p*-value: 0.010) Cramer’s V: 0.417 (*p*-value: 0.007)
Yes	33 (80.5)	6 (75)	0	39 (78)
No	1 (2.4)	2 (25)	1 (100)	4 (8)
I am not sure	7 (17.1)	0	0	7 (14)

**Appendix E.** Reliability assessment of the tool.

Reliability assessment of the tool		
Sub-scale	Cronbach’s alpha	Internal consistency
AC, Workforce	α = 0.67	Moderate
AC, Practice	α = 0.70	Acceptable
AC, Science	α = 0.60	Moderate
QA, Workforce	α = 0.49	Poor
QA, Practice	α = 0.72	Acceptable
QA, Science	α = 0.43	Poor
**Overall**	**α** **=** **0.85**	**High**

**Appendix F.** Policy/implementation framework for strengthening AC and QA in pharmacy education.

Policy/Implementation Framework for Strengthening AC and QA in Pharmacy Education	
Inputs	National PW representative body Workforce data systems (national pharmacist demand and supply) Funding for faculty development and QA standardisation Stakeholder engagement mechanisms (universities, professional associations, policymakers)
Processes	Align enrolment planning with workforce data Implement faculty development and continuous professional education programmes Standardise QA frameworks across institutions Establish formal stakeholder consultation and programme review processes Track graduate employment and workforce placement
Outputs	Evidence-based enrolment targets Trained faculty and practitioner-educators Harmonised QA standards implemented in all institutions Documented stakeholder engagement in curriculum and policy decisions Graduate tracking database operational
Outcomes	Enhanced educational capacity (DG1) Workforce supply aligned with national health system needs (DG3) Improved quality of pharmacy education and graduate readiness Sustainable pharmacy workforce development
Minimal National Indicators	Percentage of schools aligning enrolment with workforce projections Percentage of faculty completing capacity-building programmes annually Percentage of institutions implementing standardised QA frameworks Number of stakeholders formally engaged in programme development Percentage of graduates tracked with employment outcomes

**Appendix G.** Timeframe for the suggested policies and future implications.

Timeframe for the suggested policies and future implications		
Timeframe	Priority actions	Example KPIs
Short-term (1–2 years)	– Establish national PW representative body – Conduct baseline assessment of workforce demand and current QA mechanisms – Initiate faculty development workshops – Standardise stakeholder consultation processes	National body established and operational Percentage of schools with baseline QA assessment completed Percentage of faculty participating in development programmes Percentage of programmes with documented stakeholder engagement
Medium-term (3–5 years)	– Align enrolment planning with workforce data – Implement unified QA standards across all institutions – Develop and operationalise graduate tracking system – Expand faculty capacity-building programmes	Percentage of schools using workforce data for enrolment planning Percentage of institutions compliant with standardised QA frameworks Percentage of graduates tracked and reported Number of faculty completing advanced training programmes
Long-term (5–10 years)	– Integrate QA and workforce data into continuous programme improvement – Achieve sustainable alignment between education outputs and national pharmacist workforce needs – Foster regional collaboration for benchmarking and best practices	Reduction in workforce shortages relative to national targets Percentage of programmes demonstrating continuous QA improvement Number of regional benchmarking initiatives completed Improvement in graduate employment outcomes and satisfaction

## References

[CIT0001] Alhaddad, M. S. (2018). Undergraduate pharmacy students’ motivations, satisfaction levels, and future career plans. *Journal of Taibah University Medical Sciences*, *13*(3), 247–253. 10.1016/j.jtumed.2018.03.004 31435331 PMC6695073

[CIT0002] Alkhateeb, F. M., Arkle, S., McDonough, S. L. K., & Latif, D. A. (2018). Review of national and international accreditation of pharmacy programs in the Gulf cooperation council countries. *American Journal of Pharmaceutical Education*, *82*(10), 5980. 10.5688/ajpe5980 30643306 PMC6325464

[CIT0003] Almarsdóttir, A. B., & Traulsen, J. M. (2005). Rational use of medicines – An important issue in pharmaceutical policy. *Pharmacy World & Science: PWS*, *27*(2), 76–80. 10.1007/s11096-005-3303-7 15999915

[CIT0004] Anderson, C., Bates, I., Beck, D., Brock, T., Futter, B., Mercer, H., Rouse, M., Wuliji, T., & Yonemura, A. (2008). The WHO UNESCO FIP pharmacy education taskforce: Enabling concerted and collective global action. *American Journal of Pharmaceutical Education*, *72*(6), 127. doi:10.1016/S0002-9459(24)00491-1 19325947 PMC2661156

[CIT0005] Anderson, C., Bates, I., Futter, B., Gal, D., Rouse, M., & Whitmarsh, S. (2010). Global perspectives of pharmacy education and practice. *World Medical & Health Policy*, *2*(1), 5–18. 10.2202/1948-4682.1052

[CIT0006] Arakawa, N., Bruno-Tomé, A., & Bates, I. (2020). A global comparison of initial pharmacy education curricula: An exploratory study. *Innovations in Pharmacy*, *11*(1), 10.24926/iip.v11i1.2093 PMC8132530 34017634

[CIT0007] Bader, L., Bates, I., Charman, W., Bruno, A., & Carrasqueira, J. (2017). *Transforming pharmacy and pharmaceutical sciences education in the context of workforce development.*

[CIT0008] Bader, L., Bates, I., Schneider, P., & Charman, W. (2017). *Transforming pharmacy and pharmaceutical sciences education in the context of workforce development*. International Pharmaceutical Federation (FIP).

[CIT0009] Barakat, M., & Sallam, M. (2025). Pharmacy workforce: A systematic review of key drivers of pharmacists’ satisfaction and retention. *Journal of Pharmaceutical Policy and Practice*, *18*(1), 2470848. 10.1080/20523211.2025.2470848 40034876 PMC11873935

[CIT0010] Bates, I., John, C., Bruno, A., Fu, P., & Aliabadi, S. (2016). An analysis of the global pharmacy workforce capacity. *Human Resources for Health*, *14*(1), 61. 10.1186/s12960-016-0158-z 27724966 PMC5057428

[CIT0011] Bates, I., John, C., Meilianti, S., & Bade, L. R. (2018). *Pharmacy workforce intelligence: Global trends report*. International Pharmaceutical Federation (FIP).

[CIT0012] Bates, I., John, C., Seegobin, P., & Bruno, A. (2018). An analysis of the global pharmacy workforce capacity trends from 2006 to 2012. *Human Resources for Health*, *16*(1), 3. 10.1186/s12960-018-0267-y 29325554 PMC5765699

[CIT0013] Behar-Horenstein, L. S., Beck, D. E., & Su, Y. (2018). Perceptions of pharmacy faculty need for development in educational research. *Currents in Pharmacy Teaching and Learning*, *10*(1), 34–40. 10.1016/j.cptl.2017.09.019 29248072

[CIT0014] Boniol, M., Kunjumen, T., Nair, T. S., Siyam, A., Campbell, J., & Diallo, K. (2022). The global health workforce stock and distribution in 2020 and 2030: A threat to equity and ‘universal’ health coverage? *BMJ Global Health*, *7*(6), e009316. 10.1136/bmjgh-2022-009316 PMC9237893 35760437

[CIT0015] Campbell, J., Dussault, G., Buchan, J. F. P.-M., Guerra-Arias, M., Leone, C., & Siyam, A. G. C. (2013). A universal truth: No health without a workforce. In *Third Global Forum on Human Resources for Health*, Recife, Brazil.

[CIT0016] Desselle, S. (2006). Much needed attention devoted to pharmacy workforce issues. *Research in Social & Administrative Pharmacy : RSAP*, *2*(3), 294–298. 10.1016/j.sapharm.2006.08.003 17138515

[CIT0017] Diallo, K., Zurn, P., Gupta, N., & Dal Poz, M. (2003). Monitoring and evaluation of human resources for health: An international perspective. *Human Resources for Health*, *1*(1), 3. 10.1186/1478-4491-1-3 12904252 PMC179874

[CIT0018] El-Jardali, F., Jamal, D., Abdallah, A., & Kassak, K. (2007). Human resources for health planning and management in the Eastern Mediterranean region: Facts, gaps and forward thinking for research and policy. *Human Resources for Health*, *5*(1), 9. 10.1186/1478-4491-5-9 17381837 PMC1839108

[CIT0019] El-Jardali, F., Makhoul, J., Jamal, D., Ranson, M. K., Kronfol, N. M., & Tchaghchagian, V. (2010). Eliciting policymakers’ and stakeholders’ opinions to help shape health system research priorities in the Middle East and North Africa region. *Health Policy and Planning*, *25*(1), 15–27. 10.1093/heapol/czp059 19948770

[CIT0020] Enemark, S., & Williamson, I. (2004). Capacity building in land administration – A conceptual approach. *Survey Review*, *37*(294), 639–650. doi:10.1179/sre.2004.37.294.639

[CIT0021] Engle, J. P., Boyer, J. G., Travlos, D. V., & Rouse, M. J. (2023). Accreditation council for pharmacy education: 2022 annual report. *American Journal of Pharmaceutical Education*, *87*(9), 100570. 10.1016/j.ajpe.2023.100570 37442438

[CIT0022] FIPEd Global Education Report. (2013). *International Pharmaceutical Federation (FIP)*.

[CIT0023] Fisher, M., Freeman, T., Mackean, T., Friel, S., & Baum, F. (2022). Universal health coverage for health equity: From principle to practice; A response to the recent commentaries. *International Journal of Health Policy and Management*, *11*(8), 1601–1603. 10.34172/ijhpm.2022.7218 35297234 PMC9808346

[CIT0024] Global Vision for Education and Workforce. (2016). *International Pharmaceutical Federation (FIP)*.

[CIT0025] Gu, D., Andreev, K.. E., Dupre, M. (2021). Major trends in population growth around the world. *China CDC Weekly*, *3*(28), 604–613. 10.46234/ccdcw2021.160 34594946 PMC8393076

[CIT0026] Hadi, M. A., & Closs, S. J. (2016). Applications of mixed-methods methodology in clinical pharmacy research. *International Journal of Clinical Pharmacy*, *38*(3), 635–640. 10.1007/s11096-015-0231-z 26659085

[CIT0027] Hepler, C. D., & Strand, L. M. (1990). Opportunities and responsibilities in pharmaceutical care. *American Journal of Hospital Pharmacy*, *47*(3), 533–543. doi:10.1093/ajhp/47.3.533 2316538

[CIT0028] Jenner, D., Hill, A., Greenacre, J., & Enock, K. (2010). Developing the public health intelligence workforce in the UK. *Public Health*, *124*(5), 248–252. 10.1016/j.puhe.2010.03.007 20400162

[CIT0029] Knott, G. J., Mylrea, M. F., & Glass, B. D. (2020). A scoping review of pharmacy preceptor training programs. *American Journal of Pharmaceutical Education*, *84*(10), ajpe8039. 10.5688/ajpe8039 33149332 PMC7596604

[CIT0030] Lebovitz, L., & Eddington, N. D. (2019). Trends in the pharmacist workforce and pharmacy education. *American Journal of Pharmaceutical Education*, *83*(1), 7051. 10.5688/ajpe7051 30894775 PMC6418852

[CIT0031] Lewis, S. (2015). Qualitative inquiry and research design: Choosing among five approaches. *Health Promotion Practice*, *16*(4), 473–475. 10.1177/1524839915580941

[CIT0032] Liu, J. X., Goryakin, Y., Maeda, A., Bruckner, T., & Scheffler, R. (2017). Global health workforce labor market projections for 2030. *Human Resources for Health*, *15*(1), 11. 10.1186/s12960-017-0187-2 28159017 PMC5291995

[CIT0033] Mann, J. E., Amerine, L. B., Waldron, K., Wolcott, M. D., & McLaughlin, J. E. (2018). Pharmacist perceptions of competency: Identifying priority areas for a competency program development at an academic medical center. *Research in Social and Administrative Pharmacy*, *14*(6), 595–602. 10.1016/j.sapharm.2017.07.008 28754424

[CIT0034] McIsaac, M., Buchan, J., Abu-Agla, A., Kawar, R., & Campbell, J. (2024). Global strategy on human resources for health: Workforce 2030 – A five-year check-in. *Human Resources for Health*, *22*(1), 68. 10.1186/s12960-024-00940-x 39363378 PMC11450981

[CIT0035] Mukhalalati, B. A., Ashour, M., & Al Noami, A. E. (2020). Examining the motivations and future career aspirations of Qatari pharmacy students and alumni: A case study. *Currents in Pharmacy Teaching & Learning*, *12*(11), 1329–1339. 10.1016/j.cptl.2020.06.003 32867931

[CIT0036] Mukhalalati, B. A., Awaisu, A., Elshami, S., Paravattil, B., Zolezzi, M., Abu-Hijleh, M., Moslih-Almoslih, A., Carr, A., Bawadi, H., Romanowski, M., Almahasneh, R., & Bacha, R. (2022). Assessment of educational needs and design of a preceptor development program for health professional education programs in Qatar. *The Journal of Continuing Education in the Health Professions*, *42*(1), e32–e43. 10.1097/CEH.0000000000000353 34174045

[CIT0037] Mukhalalati, B. A., Bader, L., Alhaqan, A., & Bates, I. (2020). Transforming the pharmaceutical workforce in the Eastern Mediterranean Region: A call for action. *Eastern Mediterranean Health Journal = La Revue De Sante De La Mediterranee Orientale = Al-Majallah Al-Sihhiyah Li-Sharq Al-Mutawassit*, *26*(6), 708–712. 10.26719/emhj.19.064 32621506

[CIT0038] Mukhalalati, B. A., Ibrahim, M. M. M. E., Al Alawneh, M. O., Awaisu, A., Bates, I., & Bader, L. (2021). National assessment of pharmaceutical workforce and education using the International Pharmaceutical Federation’s global development goals: A case study of Qatar. *Journal of Pharmaceutical Policy and Practice*, *14*(1), 22. 10.1186/s40545-021-00305-y 33612105 PMC7898757

[CIT0039] Nanjing Statements: Statements on Pharmacy and Pharmaceutical Sciences Education. (2017). *International Pharmaceutical Federation (FIP).*

[CIT0040] Narasimhan, V., Brown, H., Pablos-Mendez, A., Adams, O., Dussault, G., Elzinga, G., Nordstrom, A., Habte, D., Jacobs, M., Solimano, G., Sewankambo, N., Wibulpolprasert, S., Evans, T., & Chen, L. (2004). Responding to the global human resources crisis. *The Lancet*, *363*(9419), 1469–1472. 10.1016/S0140-6736(04)16108-4 15121412

[CIT0041] Nkansah, N., Mostovetsky, O., Yu, C., Chheng, T., Beney, J., Bond, C. M., & Bero, L. (2010). Effect of outpatient pharmacists’ non-dispensing roles on patient outcomes and prescribing patterns. *The Cochrane Database of Systematic Reviews*, *2010*(7), CD000336. 10.1002/14651858.CD000336.pub2 20614422 PMC7087444

[CIT0042] Pharmaceutical Workforce Development Goals. (2016). *International Pharmaceutical Federation (FIP)*.

[CIT0043] Quality Assurance of Pharmacy Education: The FIP global framework. (2014). *International Pharmaceutical Federation (FIP)*.

[CIT0044] Ramli, A., & Mohd Zain, R. (2019). The impact of facilities on student’s academic achievement. *Sci.Int.(Lahore)*, *30*(2), 299–311.

[CIT0045] Rees, G. H. (2015). Addressing inadequate health workforce intelligence. *Internal Medicine Journal*, *45*(9), 887–889. 10.1111/imj.12847 26332620

[CIT0046] Robinson, P. (2007). Designing and conducting mixed methods research. *Australian and New Zealand Journal of Public Health*, *31*(4), 388. 10.1111/j.1753-6405.2007.00096.x

[CIT0047] Rueben, A., Forsyth, P., & Thomson, A. H. (2020). Professional development beyond foundation training: A study of pharmacists working in Scotland. *International Journal of Pharmacy Practice*, *28*(2), 165–172. 10.1111/ijpp.12585 31583789

[CIT0048] Service, R. (2009). Book Review: Corbin, J., & Strauss, A. (2008). Basics of qualitative research: Techniques and procedures for developing grounded theory (3rd ed.). Thousand Oaks, CA: Sage. *Organizational Research Methods*, *12*(3), 614–617. 10.1177/1094428108324514

[CIT0049] Taber, K. S. (2018). The use of Cronbach’s alpha when developing and reporting research instruments in science education. *Research in Science Education*, *48*(6), 1273–1296. 10.1007/s11165-016-9602-2

[CIT0050] Tavakol, M., & Dennick, R. (2011). Making sense of Cronbach’s alpha. *International Journal of Medical Education*, *2*, 53–55. 10.5116/ijme.4dfb.8dfd 28029643 PMC4205511

[CIT0051] The FIP Development Goals report 2021: Setting goals for the decade ahead. (2022). *International Pharmaceutical Federation (FIP)*.

[CIT0052] Tong, A., Sainsbury, P., & Craig, J. (2007). Consolidated criteria for reporting qualitative research (COREQ): A 32-item checklist for interviews and focus groups. *International Journal for Quality in Health Care: Journal of the International Society for Quality in Health Care*, *19*(6), 349–357. 10.1093/intqhc/mzm042 17872937

[CIT0053] Toyon, M. (2021). Explanatory sequential design of mixed methods research: Phases and challenges. *International Journal of Research in Business and Social Science (2147- 4478)*, *10*(5), 253–260. 10.20525/ijrbs.v10i5.1262

[CIT0054] Van den Bogert, C., Mestrinaro, M., & Weerasuriya, K. (2012). *The pursuit of responsible use of medicines: Sharing and learning from country experiences.*

[CIT0055] WHO’s Strategy for the Eastern Mediterranean Region, 2020–2023: Turning Vision 2023 into action. (2019). *WHO Regional Office for the Eastern Mediterranean*.

[CIT0056] Winit-Watjana, W. (2016). Research philosophy in pharmacy practice: Necessity and relevance. *The International Journal of Pharmacy Practice*, *24*(6), 428–436. 10.1111/ijpp.12281 27339891

[CIT0057] Zeitoun, A., Sacre, H., Hallit, S., Zeenny, R. M., Sili, G., & Salameh, P. (2020). Clinical preceptor competencies for a better pharmacy education: A suggested framework for Lebanon. *Journal of Pharmaceutical Policy and Practice*, 13(1), 21. 10.1186/s40545-020-00217-3 32587705 PMC7313193

